# An interaction mechanism for the maintenance of fission–fusion dynamics under different individual densities

**DOI:** 10.7717/peerj.8974

**Published:** 2020-05-14

**Authors:** David Bierbach, Stefan Krause, Pawel Romanczuk, Juliane Lukas, Lenin Arias-Rodriguez, Jens Krause

**Affiliations:** 1 Department of Biology and Ecology of Fishes, Leibniz-Institute of Freshwater Ecology and Inland Fisheries, Berlin, Germany; 2 Faculty of Life Sciences, Thaer Institute, Humboldt Universität Berlin, Berlin, Germany; 3 Department of Electrical Engineering and Computer Science, Lübeck University of Applied Sciences, Lübeck, Germany; 4 Department of Biology, Institute for Theoretical Biology, Humboldt Universität Berlin, Berlin, Germany; 5 Bernstein Center for Computational Neuroscience, Humboldt Universität Berlin, Berlin, Germany; 6 División Académica de Ciencias Biológicas, Universidad Juárez Autónoma de Tabasco, Villahermosa, Mexico

**Keywords:** Poecilia, Markov chain, Social network analysis, Cave fish, Fission–fusion society

## Abstract

Animals often show high consistency in their social organisation despite facing changing environmental conditions. Especially in shoaling fish, fission–fusion dynamics that describe for which periods individuals are solitary or social have been found to remain unaltered even when density changed. This compensatory ability is assumed to be an adaptation towards constant predation pressure, but the mechanism through which individuals can actively compensate for density changes is yet unknown. The aim of the current study is to identify behavioural patterns that enable this active compensation. We compared the fission–fusion dynamics of two populations of the live-bearing Atlantic molly (*Poecilia mexicana*) that live in adjacent habitats with very different predator regimes: cave mollies that inhabit a low-predation environment inside a sulfidic cave with a low density of predatory water bugs (*Belostoma* sp.), and mollies that live directly outside the cave (henceforth called “surface” mollies) in a high-predation environment. We analysed their fission–fusion dynamics under two different fish densities of 12 and 6 fish per 0.36 m^2^. As expected, surface mollies spent more time being social than cave mollies, and this difference in social time was a result of surface mollies being less likely to discontinue social contact (once they had a social partner) and being more likely to resume social contact (once alone) than cave mollies. Interestingly, surface mollies were also less likely to switch among social partners than cave mollies. A random walk simulation predicted each population to show reduced social encounters in the low density treatment. While cave mollies largely followed this prediction, surface mollies maintained their interaction probabilities even at low density. Surface mollies achieved this by a reduction in the size of a convex polygon formed by the group as density decreased. This may allow them to largely maintain their fission–fusion dynamics while still being able to visit large parts of the available area as a group. A slight reduction (21%) in the area visited at low densities was also observed but insufficient to explain how the fish maintained their fission–fusion dynamics. Finally, we discuss potential movement rules that could account for the reduction of polygon size and test their performance.

## Introduction

In many group-living animals, individuals switch frequently between social and solitary periods (Krause & Ruxton, 2002). This type of social interaction is wide-spread and known as fission–fusion dynamics (Aureli et al., 2008; Couzin & Laidre, 2009). Anybody who has ever observed fish will have noticed the extremely frequent fission–fusion dynamics at the rate of a few seconds that many species display. The duration of these associations between individuals follows a geometric distribution (i.e., with short associations being very frequent and long ones very rare, see Wilson et al., 2015b; Wilson et al., 2015a). It has been argued that a geometric distribution could be a good strategy in response to constant predator threats: if a predator is almost always present and monitors prey for moments when it is alone then an association dynamic that follows a geometric distribution could be adaptive because there would be no typical period for which the predator has to wait for the prey to be alone (see Wilson et al., 2015b; Scannell, Roberts & Lazarus, 2001; Bednekoff & Lima, 2002 for a discussion). The underlying idea is that the maintenance of geometric fission–fusion dynamics combines the benefits of group-living (e.g., to lower an individual’s risk of predation) with those of being solitary (e.g., lower resource competition) without becoming predictable to predators.

Maintaining population-specific fission–fusion dynamics under different environmental or social conditions is probably highly advantageous. Experiments in the live-bearing guppy (*Poecilia reticulata*) found fish to keep their social dynamics even when observed over several years and when translocated to different habitats. Thus, these fish compensate changes in conditions like group density or pool size by somehow actively maintaining a certain social dynamic (Wilson et al., 2015b; Wilson et al., 2014). While social rank order (Hobson & DeDeo, 2015; Pinter-Wollman et al., 2013) or the ‘inheritance’ of social bonds from parents to offspring (Ilany & Akçay, 2016) may explain consistency in social dynamics in species with long-lasting social bonds, for shoaling or herding species with shorter social bonds, no mechanism has been proposed yet.

A possible starting point to gain insights into the mechanism of these compensatory abilities may be to compare populations that possess these abilities to those that do not. We hypothesised that animals living under high predation pressure and for which the maintenance of fission–fusion dynamics is most beneficial should be able to compensate for changing conditions. For animals living in almost predator-free habitats, costs of group-living may outweigh its benefits (Marino, 2010), and thus these animals may have lost or never acquired the ability to actively maintain certain fission–fusion dynamics under varying environmental conditions.

Due to the fact that virtually no habitat can be considered as entirely free of predators, we based our investigation within a study system that provides both the very rare case of a population that evolved in the absence of any visually hunting predators, as well as an adjacent population that experiences extremely high rates of visually hunting predators. In southern Mexico, an ancestral form of the Atlantic molly (*Poecilia mexicana*; in the following referred to as ‘molly’) colonised sulfidic springs as well as sulfidic cave environments about 100,000 years ago (Riesch, Tobler & Plath, 2015; Tobler et al., 2018; Pfenninger et al., 2014). The sulfidic water is toxic to non-adapted metazoans and thus mollies are the only fishes that are found residing permanently in the H_2_S-rich waters (Greenway et al., 2014; Bierbach et al., 2013; Bierbach et al., 2011). Besides its toxicity, H_2_S further depletes the water from oxygen and thus both cave and surface mollies swim most of their time near the water surface to skim the thin but oxygen-rich surface layer (Plath et al., 2007). In case of the surface mollies, this behaviour attracts fish-eating birds and rates of bird predation are much higher compared to non-sulfidic surface streams in the same area (Riesch et al., 2010). For the cave molly, the aquatic surface respiration behaviour does not lead to the same attraction of predators, as the absence of light prevents piscivorous birds from hunting inside the cave. In fact, cave-dwelling mollies, that still possess functional albeit smaller eyes (Körner et al., 2006), are only preyed on by water bugs and spiders, for which exclusively tactile prey detection is assumed (see Tobler, 2009; Klaus & Plath, 2011; Horstkotte et al., 2010). Thus, the cave environment can be assumed to be free of visually hunting predators (Bierbach et al., 2013; Bierbach et al., 2011). Interestingly, earlier laboratory works found cave mollies to be less social compared to their surface-dwelling counterparts (Plath & Schlupp, 2008; Bierbach et al., 2018; Riesch et al., 2006) that live only a few meters away outside the cave and share the same H_2_S-containing water. We thus predicted that cave mollies (1) are less social when observed in their natural habitats, and (2) due to the lack of predator exposure, cave-dwelling mollies may not possess the ability to keep their social dynamics constant across different densities. If so, cave mollies may be seen as a natural ‘null model’, i.e., individuals may follow movement or interaction rules that resemble those of ‘random walk/interaction models’ which are often used in social network analysis to evaluate the significance of observed animal interaction patterns (Croft et al., 2011).

To test our initial predictions, we manipulated densities of mollies from both populations that were kept in a net cage in their respective habitat. Groups were videotaped and positional information was extracted through semi-automated video tracking. The resulting movement and association of all group members were analysed using the Markov chain model of Wilson et al. (2014), which has been applied to the social systems of the closely related guppy (Wilson et al., 2015b; Borner et al., 2015). It describes the social behaviour commonly displayed by all focal individuals as sequences of ‘behavioural states’ and provides the probabilities of fish switching between these states (see ‘Methods’ for more details). In contrast to previous studies on shoaling behaviour, which usually looked only at the percentage of time spent being social (Plath & Schlupp, 2008; Bierbach et al., 2018), the above approach allows us to decompose the social interactions into separate behavioural states which offers a more detailed insight into how selection has acted on social behaviour.

Taking the Markov chain modelling approach, we can also predict how changes in individual densities will affect fission–fusion dynamics, unless the fish work actively against them (Table 1). To do so, we used a ‘random walk simulation’ introduced by Wilson et al. (2014) (see also section Modelling of fission–fusion dynamics below for details). If fish density decreases, the simulation predicts that encounter probabilities between fish should also decrease. As a consequence, solitary phases should increase in length. At the same time, the length of social phases should decrease because after leaving a social partner the chances of being alone will be higher. Overall, the length of social periods should decrease with decreasing density, while the length of contact phases with the same social partner should remain constant. Consequently, with a decrease in time being social the number of switches of social partners should also decrease. We predict that cave mollies should largely follow the above predictions in model probabilities when density is decreased, while surface mollies should be able to somehow compensate for density changes and maintain their fission–fusion dynamics as has been the case for other surface-dwelling fishes (Wilson et al., 2014).

**Table 1:** Specific predictions for social state probabilities (see main text) comparing surface and cave-dwelling mollies (*Poecilia mexicana*).

Model probabilities	Comparative prediction
probability of ending social contact	Surface < Cave
probability to join a new social partner once they are alone	Surface > Cave
probability of leaving a current nearest neighbour	Surface < Cave
probability of switching social partners	Surface < Cave

If we found differences between surface and cave dwelling mollies in compensatory ability, we aimed to explore different mechanisms by which this could be achieved. By combining our fine-scaled movement data for all group members at different densities with a random walk simulation, we can evaluate which behavioural parameters are actively changed during the density compensation. We explored three different behavioural aspects that can be responsible for the assumed density compensation of fission–fusion dynamics: (a) fish might swim at higher speeds, (b) fish might reduce the area usage to a particular part of the experimental arena, and (c) the polygon formed by the group (i.e., the convex hull that encloses all individuals of a group) might decrease while allowing fish to explore most of the arena.

## Methods

### Study system

**Figure 1:**
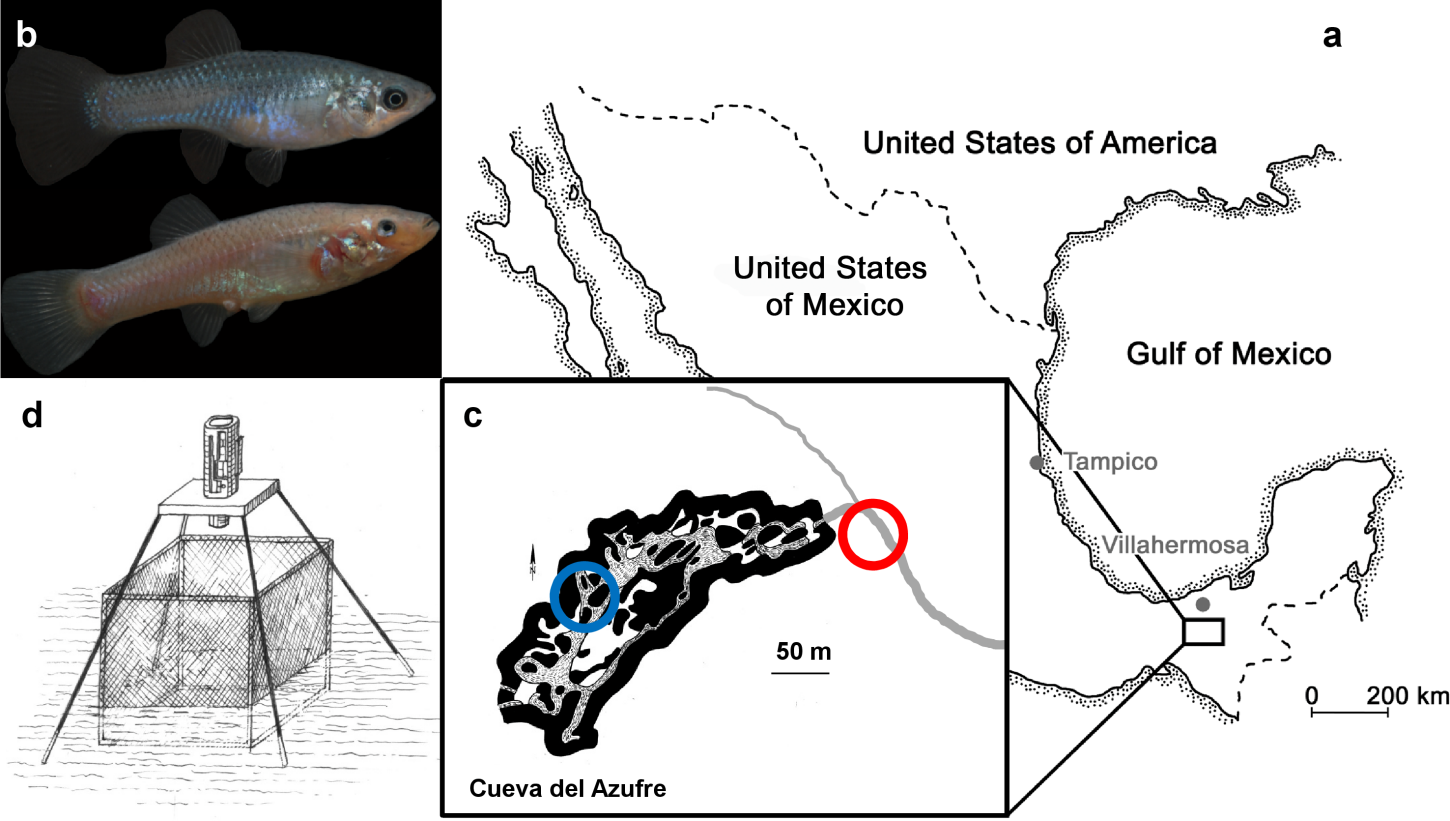
The study system. (A)**** Both tested molly populations originate from the South of Mexico near the city of Tapijulapa, federal state of Tabasco. Here, ancestral forms of *Poecilia mexicana* colonized both surface; (B, top, picture of a surface-dwelling molly) as well as cave; (B, bottom, picture of cave molly) habitats. (C) Locations of the study sites are indicated with blue (cave) and red (surface) circles along the El Azufre river that flows from left to right, i.e., out of the cave. On site, individually marked fish were transferred into a net cage and recorded with an infrared camera (D).

Our study sites are located near the Mexican city of Tapijulapa (Fig. 1, Tabasco, United States of Mexico). Here, the Río Tacotalpa and its tributaries drain through the mountains of the Sierra Madre of Chiapas and several sulphide spring complexes of volcanic origin have been discovered in the foothills of the Sierra Madre. This area is also rich on natural cave formations, two of which are known to harbour endemic populations of the otherwise widely distributed Atlantic molly, *Poecilia mexicana* (the so-called ‘cave molly’; Fig. 1B; see Parzefall, 2001; Gordon & Rosen, 1962; Tobler et al., 2008). The Cueva del Azufre (aka Cueva del Sardinia or Cueva Villa Luz) is divided into 13 different chambers, with Chamber XIII being the innermost chamber (after Gordon & Rosen, 1962), see map of the cave in Fig. 1). The front chambers obtain some dim light through natural skylights in the cave’s ceiling, whereas the rearmost cave chambers (from Chamber VI onwards) lay in complete darkness. Several springs in the cave (mainly in Chamber X) release sulfidic water, and the creek that flows through the cave eventually leaves the underground and turns into the sulfidic El Azufre River. H_2_S concentrations in the cave as well as in the EL Azufre river are high (23 µM to 320 µM,) and accompanied with very low oxygen levels (<1.5 mg/L, see Tobler et al., 2006). The surface form of the Atlantic molly (Fig. 1B) is widespread in freshwaters along the Central American Atlantic coast (Miller, 2006) and independently colonised sulfidic springs and rives, of which the El Azufre River is one (Tobler et al., 2018).

### Experimental setup

#### Overview

We compared the fission–fusion dynamics of sulphide-adapted surface mollies (in light) with those of sulphide-adapted cave mollies (in total darkness) under two different fish densities. We caught and marked fish from the El Azufre river and in cave chamber VII (see Fig. 1B). Afterwards we introduced small groups of fish (*N* = 12 individuals per group, equal sex ratio) into a custom made net cage that was placed at the respective site (Fig. 1C). After one day of acclimation, we filmed these groups of 12 individuals for 45 min (hereafter treatment “G12a”) with a camera under infrared light as *P. mexicana* does not possess infrared-sensitive photoreceptors (Körner et al., 2006). In order to compare the fission–fusion dynamics of both populations when fish density was changed, we removed 6 fish from the arena after the initial videotaping and the remaining 6 individuals (3:3 male:female ratio) were videotaped again (hereafter treatment “G6”). Afterwards, the 6 previously removed individuals were reintroduced and we videotaped the group of 12 fish another time (hereafter treatment “G12b”). We carried out 3 replicates for the cave mollies and 2 replicates for the surface mollies.

This 12-6-12 design was used to see whether the fission–fusion dynamics went back to normal after the removed fish were transferred back into the arena. We discarded the first 35 min of each video to provide fish ample time to recover from the handling disturbances before scoring their behaviour (more details below). We then extracted positional information for fish and built social networks through a Markov chain approach (see Wilson et al., 2014). We are aware that our treatments altered both number of individuals per group as well as individuals per area in the observational arena. However, for reasons of clarity we will refer to the change in individual numbers in the arena (G12a to G6 to G12b) as density change.

#### Individual tagging and housing of experimental fish before the recordings

Upon capture, fish were tagged immediately on site using VIE colours (see Bierbach et al., 2014 for similar approach in the laboratory). To do so, fish were anesthetised with clove oil and carefully injected with VIE colours at different locations along their dorsal surface. VIE colours are visible in both day-light and infra-red light (as bright white spots) and this allowed us to individually recognise our fish in both environments. After the tagging, we transferred 12 individuals (6 males, 6 females) into a cubic net cage (60 cm by 60 cm by 60 cm; mesh-width 2 mm; Fig. 1D and Video S1). At both sites, the cage was placed in a shallow area with a water depth of about 5 cm and the net bottom was covered entirely with natural gravel found around the cage. Fish were left in the cage overnight for acclimatisation and a constant but slow water through-flow ensured food items reaching the cage.

#### Video recording of social networks

The next morning we filmed the fish with a full HD camera (Canon XF200, recording at 50 fps) which was able to record both in infrared light (used in the cave with an additional IR light; Dedolight Redzilla DLOBML-IR860, 860 nm) and normal light (used outside the cave in the El Azufre river). The camera was fixed centrally above the cage with a custom-made tripod (Fig. 1D). To limit surface reflections due to water movements hampering with tracking, we closed any inflow into the cage by covering the outside walls with plastic foil during the experimental recordings. In the cave, we minimized disturbances during fish handling by using small head lights that were covered with red foil (see Riesch et al., 2016 for a similar approach when rearing cave fish in the laboratory). Once recordings were started, all experimenters left the site to avoid disturbances. For the removal of fish during the G6 treatment, we haphazardly selected and removed 3 males and 3 females from the cage. These fish were put into a similar net cage ca. 5 m downstream prior to the G12b treatment. This procedure reduced the number of fish inside the cage by 50% (G6) and was inspired by experiments on Trinidadian guppies in natural ponds (Wilson et al., 2015b). After the last recording (G12b), we measured body size (standard length, SL) of all experimental fish to the nearest millimetre. The body lengths of cave and surface mollies did not differ (body length mean ± SE: cave mollies = 37 mm  ± 1.1, surface mollies = 38 mm ± 1.4, *p* = 0.7 in a two samples *t*-test).

### Modelling of fission–fusion dynamics

From all video recordings (3 videos per group, G12a, G6 and G12b), we analysed a footage of 3 min, which started 35 min after the recording was initialised. Pre-trials found fish in the cage to behave naturally ca. 10 min after introduction and by conservatively extending this period to 35 min we ensured to observe undisturbed social interactions. We analysed only 3 min of the remaining 10 min of each 45-min video, because as experimenters returned to the cave to stop the recording and initiate the next treatment, they might have illuminated parts of the chamber with the head lamps and thus disturbed the fish.

For those 3 min of footage, we individually tracked the positions of all fish using the video tracking software Ethovision XT10. Automated detection and tracking of individual fish was difficult due to the low contrast in the videos and tracks had to be corrected manually frame by frame. Although videos were recorded at 50 fps, we sampled fish positions at a constant rate of 5fps. As a result, we gained 900 X-Y-positions per individual and treatment, which we used in our analyses of swimming distances, area usage, and polygon size utilizing custom-made software. For our Markovian models we used a subset of approximately 40 data points per individual and treatment (see the next section for details) which is similar to previous studies employing Markovian Models for social network analysis in fishes (Wilson et al., 2015b).

#### The Markovian Model

As a basis for the investigation of the fission–fusion dynamics of the mollies we used the stochastic model of Wilson et al. (2014) (see Fig. S1). It can explain a number of aspects of fission–fusion dynamics, including the percentage of time individuals are social (or alone), the number and lengths of contact phases between individuals, as well as the number and lengths of solitary phases.

The model can be characterised by specifying the 3 probabilities of leaving the current nearest neighbour (*P*_leave_nn_), of discontinuing social contact in general (*P*_s→a_), and of discontinuing being alone (*P*_a→s_). The reciprocal values of these probabilities are proportional to the mean lengths of contact phases with the same neighbour, of phases of being social (with any neighbour), and of phases of being alone, respectively. In addition, the probability of switching social partners within the period of social time (*P*_switch_nn_) can be derived from the model (see Text SI for details on the model).

The data points for the construction of this model were collected by observations of focal individuals. Every *t* seconds the nearest neighbour of the focal individual was recorded, where two individuals were regarded as being neighbours of each other, if their distance was smaller than a value *d*. If no conspecific was within a radius of *d*, the individual was regarded as being alone. Wilson et al. (2014) chose *d* = 4 body lengths and *t* = 10 s for their study on guppies. The mollies in our study system tended to be closer together, and we used eight cm (ca. 2 body lengths) as a value for *d*. As a value for *t* we used 5 s, although our data provide a much higher time resolution. This seemed to be a sensible choice, because the fish rarely changed their behaviour regarding the modelled aspects at a higher rate. Also, if each frame of the raw tracking data was used, qualitative misinterpretations could occur because very short phases of two fish being close to each other (for example, when passing from different directions) could be mistaken for a contact phase. We confirmed that our results were robust regarding other choices for *d* and *t* (see Fig. S2). The model probabilities can be estimated based on relative frequencies. Following Wilson et al. (2014) we did not take the specific individual identities into account when estimating the model probabilities, i.e., we counted the state changes regardless of the individual identities across all groups. We used the model to describe the general dynamics commonly displayed by all observed individuals for each treatment.

We compared model probabilities by looking at their 95% confidence intervals, which we computed using the function binom.test in R (R Core Team, 2013). In our study, we have to be careful, because all focal individuals were observed simultaneously. This means, each contact phase was observed twice (once for each individual as a focal individual). To make sure that the confidence intervals of the model probabilities are not biased, we divided the numbers of data points used for their computation by 2.

#### The random walk simulation

To quantify the expected effects of changes in density on fission–fusion dynamics, we performed a simulation of random movements of individuals, which has already been used by Wilson et al. (2014). In this simulation the individuals move independently of each other, following the same simple pattern, respectively. In particular, between two successive time points each individual moves by a fixed distance and with a certain probability changes its heading (see Text S2 for a detailed description). By changing the number of individuals while keeping all other parameters of the movement simulation constant, we determined the relative magnitude of changes to the measures under investigation (e.g., Markovian probabilities, area usage and polygon reduction; for the latter two, please see description below). We extracted all values from the simulation in the same way we extracted them from our tracked videos.

#### Exploring possible mechanisms of active density compensation

(1) Swimming distances (speed) in cave and surface-dwelling mollies

In our analysis of individual swimming distances we included only those 6 individuals of each group that were present in all 3 treatments. To compare the swimming distances between two treatments, we computed the mean individual swimming distance of each treatment. As most measures taken from the behaviour of individuals in a group are dependent (Krause, Croft & James, 2007), we used a randomisation test, where the absolute value of the difference of the mean swimming distances constituted the test statistic. In each randomisation step the values of each individual were swapped between the treatments with a probability of 0.5. In other words, the observed values for each individual were randomly assigned to the treatments. For each test we performed 10^5^ randomisation steps.

(2) Area usage in cave and surface-dwelling mollies

To measure the group area use, we divided the total area (60 ×  60 cm^2^) into 16 squares of 15 × 15 cm^2^ and counted the number of squares visited by some individual of the group. These values were then compared to our random walk simulation. This approach provides evidence on whether a reduction in area usage would be sufficient to maintain the observed probabilities of fission–fusion dynamics for G6 and thus account for the density compensation.

(3) Size of the polygons formed by the groups of cave and surface-dwelling mollies

We computed the mean perimeter and mean area of the convex polygons formed in the three treatments. In contrast to the analyses of individual measures, we took the complete group into account and not just the 6 individuals from the G6 treatments. To estimate the magnitude of this effect, we computed the mean perimeter and mean area from the random walk simulations used for the analysis of the fission–fusion dynamics. In order to test the significance of these reductions, we determined the 0.025 percentiles of the distribution of mean perimeters and mean areas using 10^4^ repetitions of our random walk simulation.

(4) Mechanisms for changes in convex polygon sizes spanning the group

Stronger than predicted changes in polygon size can be due to individual-based movement rules that allow the fish to actively compensate for density changes. If fish do not swim faster or reduce their area usage, the reduction in polygon size could be a result of a tendency to move back into the polygon whenever a fish is at a vertex of the convex polygon and without a neighbour within its social radius (two body lengths in our case). We implemented this rule in our random walk simulation (choosing a probability of 80% to model the tendency to follow the rule) and compared polygon size reduction with those obtained by real fish groups and random walk simulations without this rule.

Beside individual-based movement rules that fish could perform to achieve changes in polygon size, the observed reduction in polygon size in surface mollies could be a result of a fundamental change in behaviour from a fission–fusion system to schooling when density changed. To investigate this issue, we measured the polarisation of the 6 fish that were present in all three treatments by computing the sum of the unit velocity vectors of these 6 individuals every 3 s. Then we computed the mean of these sums. The interval of 3 s was chosen, because sometimes fish did not move for almost 3 s, leading to an undefined unit vector. The sum of the vectors is always in the range 0–1. The value 1 occurs if all fish swim in exactly the same direction, whereas the value 0 occurs if all swimming directions cancel each other out.

### Ethics

Experiments reported in this study were carried out in accordance with the recommendations of “Guidelines for the treatment of animals in behavioural research and teaching” (published in Animal Behaviour 1997) and under the authorisation of the Mexican government (H. Ayuntamiento Constitucional Tacotalpa, Direction Fomento Economico y Turismo; DGOPA.09004.041111.3088, PRMN/DGOPA-003/2014, PRMN/DGOPA-009/2015, and PRMN/DGOPA-012/2017, issued by SAGARPA-CONAPESCA-DGOPA).

## Results

### Fission–fusion dynamics of cave and surface mollies

**Figure 2:**
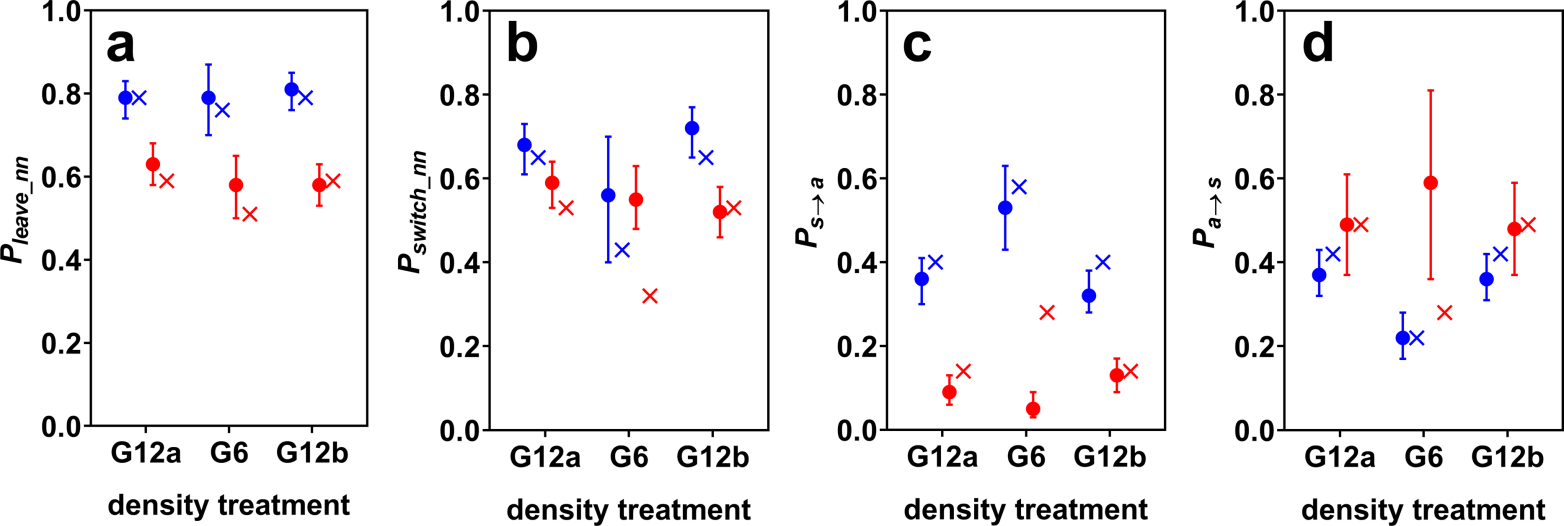
Estimated model probabilities (plus 95% confidence intervals) for cave (blue) and surface mollies (red). Shown are (A) probabilities of leaving the current nearest neighbour (*P*_leave_nn_), (B) of switching social partners within the period of social time (*P*_switch_nn_), (C) of discontinuing social contact in general (*P*_s→a_), and (D) of discontinuing being alone (*P*_a→s_). The probabilities resulting from a random walk simulation are marked by x’s. The parameters of the simulation were chosen such that it roughly reproduced the observed model probabilities of the groups of 12 fish (see main text).

Surface mollies showed lower probabilities of leaving the current social partner and of leaving any social partner than cave mollies (Fig. 2). We found a similar trend for the probability of switching between social partners in the two G12 treatments. The similarity of the switching probabilities for G6 is probably coincidental and only caused by cave mollies not actively working against density changes (see next paragraph). Surface mollies also showed a higher probability to join a new social partner once they were alone compared to cave mollies (but this difference was less pronounced for G12 than for G6, Fig. 2).

### Effect of density changes on cave molly fission–fusion dynamics

The fission–fusion dynamics of cave mollies changed with changing density (from G12a to G6) as predicted by the random walk simulation. The probabilities *P*_a→s_ and *P*_switch_nn_ decreased while *P*_s→a_ increased, when the density decreased, i.e., the lengths of phases of being alone increased and the lengths of social phases decreased. The confidence intervals of *P*_s→a_ for the two treatments with 12 fish (G12a and G12b) were very similar but did not overlap with the confidence interval for the 6 fish treatment (G6). The same holds for *P*_a→s_. As predicted, *P*_leave_nn_ did not seem to be influenced by density changes either, i.e., the length of contact phases with the same social partner did not differ between G12 and G6.

The simulation predicted not only the trend of changes in fission–fusion dynamics, but also approximately its magnitude. We chose the simulation parameters to roughly reproduce the fission–fusion dynamics observed with 12 fish (G12a) and then ran the simulation for 6 fish, which generally reproduced the fission–fusion dynamics observed with the live fish during G6 (Fig. 2). The percentage of overall time spent being social dropped from 52% (51% in the simulation) for the groups of 12 fish to 29% (27% in the simulation) for groups of 6 fish. This suggests that cave mollies followed the null model as expected.

### Effect of density changes on surface molly fission–fusion dynamics

For surface mollies the results were markedly different. The confidence intervals of all model probabilities overlapped between the treatments with 12 and with 6 fish (Fig. 2). Also, the observed probabilities in the treatment with 6 fish differed considerably from the predictions of the simulation (Fig. 2). The percentage of overall time spent being social increased from 81% (78% in the simulation) for the groups of 12 fish to 92% (50% in the simulation) for groups of 6 fish. This suggests that surface mollies worked actively against density changes in order to maintain their fission–fusion dynamics.

### Exploring mechanism of active density compensation

(1) Swimming distances in cave and surface-dwelling mollies

Swimming distances of cave mollies did not change with density (Tables 2 and 3). This is in line with the results regarding their fission–fusion dynamics, which suggested that cave mollies are not able to maintain their fission–fusion dynamics under reduced densities. Surface mollies, however, on average swam longer distances in groups of 12 than in groups of 6, although only the difference between G12a and G6 was significant (Tables 2 and 3). This result shows that the maintenance of sociality of surface mollies in the G6 treatment could not be explained by a proposed greater swimming distance (i.e., higher swimming speeds) during G6.

**Table 2:** Mean individual swimming distances of the 6 fish that were present in all three treatments and mean area usage of the complete groups of the cave (a) and the surface mollies (b). The area usage indicates the percentage of squares visited in a 4 × 4 grid.

	(a) Cave molly		(b) Surface molly	
Treatment	individual swimming distance (cm)	group area usage	individual swimming distance (cm)	group area usage
G12a	662	100%	355	97%
G6	624	98%	256	78%
G12b	572	100%	320	100%

**Table 3:** Results of randomisation tests (*N* = 105 repetitions) regarding differences in individual swimming distances for the cave (a) and the surface mollies (b). Significant results are marked with star.

	(a) Cave molly		(b) Surface molly	
Compared treatments	Value of test statistic	*P*-value	Value of test statistic	*P*-value
G12a - G12b	89.2	0.13	35.1	0.18
G12a - G6	37.8	0.58	99.2	<0.01*
G12b - G6	51.4	0.18	64.1	0.12

(2) Area usage in cave and surface-dwelling mollies

The group area usage of cave mollies did not change with density (Table 2), which again suggests that cave mollies did not compensate for density changes. Surface mollies, however, reduced their group area usage on average by 21% when the number of fish decreased. Using our random walk simulation, we tested whether this reduction would be sufficient to maintain the observed probabilities of fission–fusion dynamics for G6 and thus account for the density compensation. Our simulation indicated that this was not the case (*P*_s→a_ observed= 0.05, simulated with area reduction = 0.24, simulated without area reduction = 0.27; *P*_a→s_ observed = 0.59, simulated with = 0.33 and without area reduction = 0.28; overall social time observed = 92% and simulated with = 58% and without area reduction = 50%). For surface mollies, an area reduction of 60% (instead of only 21%) would be necessary to achieve the observed probabilities of fission–fusion dynamics for G6.

(3) Size of the polygons formed by the groups of cave and surface-dwelling mollies

The behaviour of cave mollies was again predicted by the simulation (Table 4). The reductions of both the mean perimeter and the mean area of the convex polygons formed by the group could be explained by a reduction in density following the null model. As expected, the results were different for the surface mollies (Table 4). Here, the reductions of both the mean perimeter and the mean area of the polygons were much larger than the density reduction alone can explain. For both measures the observed values (55% reduction of the perimeter and 86% reduction of the area) were greater than the 0.025 percentiles (25% reduction of the perimeter and 56% reduction of the area). Therefore, the size reduction of the polygons can be regarded as significant.

**Table 4:** Mean perimeter and mean area of the convex polygons formed by the groups in the three treatments of the cave (a) and the surface mollies (b). For the groups of size 6 the reduction relative to the values of the groups of size 12 is shown. The simulation results are the mean results from 1,000 random walks. The values marked with an asterisk were obtained from a simulation with the movement rules explained in Fig. 3.

	Mean perimeter [cm]	%reduced		Mean area [cm^2^]	%reduced	
	observed	observed	simulated	observed	observed	simulated
(a) Cave molly						
G12a	187			2,172		
G6	157	15	17	1,285	38	42
G12b	181			1,977		
(b) Surface molly						
G12a	103			620		
G6	49	55	17 (39)*	93	86	42 (70)*
G12b	112			698		

(4) Causes for polygon size changes in surface mollies

**Figure 3:**
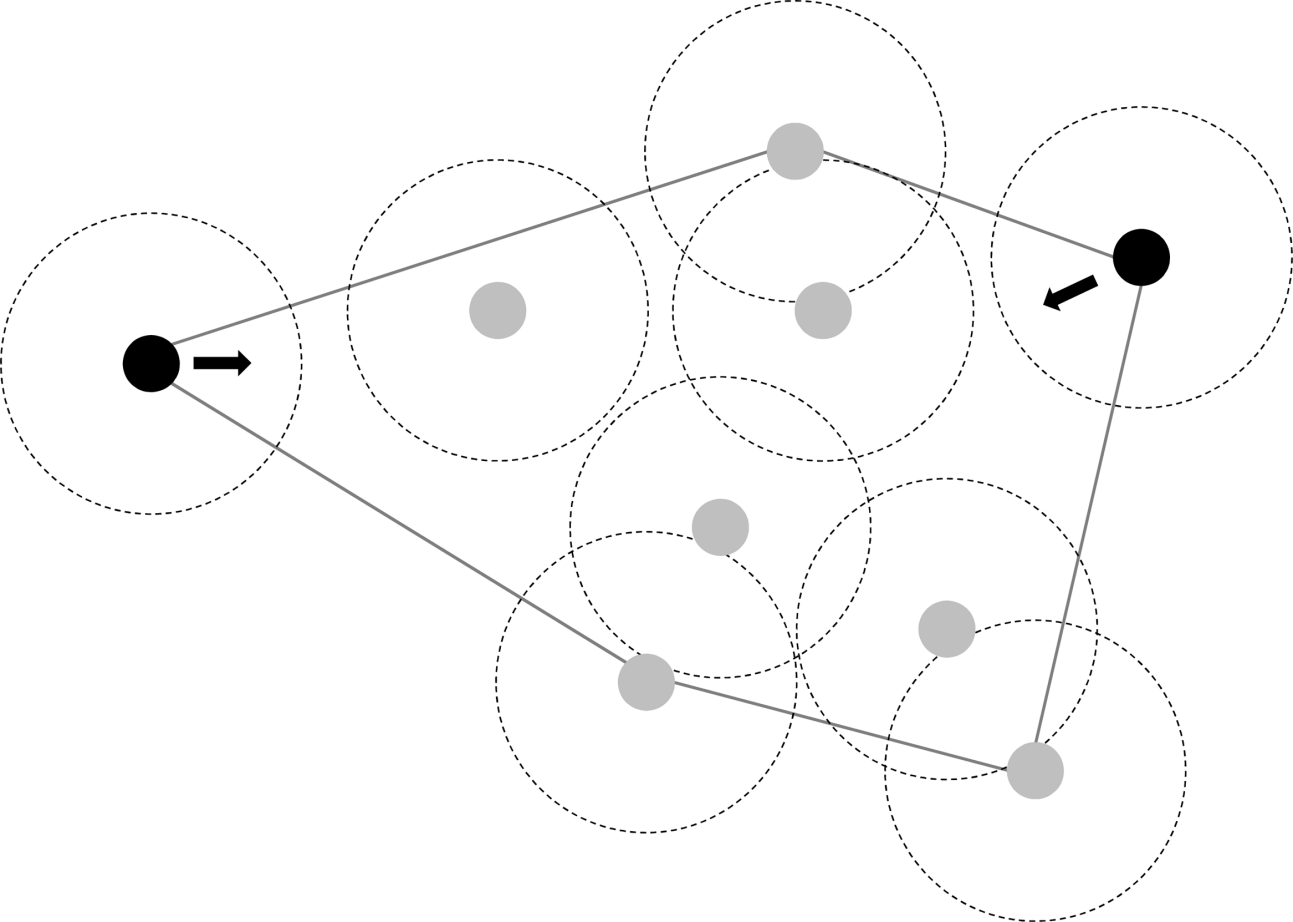
Illustration of individual movement rules of fish which could largely explain observed changes in polygon size (in response to density changes) and maintain fission-fusion dynamics. Individuals at the vertex of a polygon without a neighbour within eight cm move at the next time step back into the polygon area (instead of further out) with a probability of 80%.

Simulating fish to move back into the polygon at the next time step whenever they were at the vertex of a convex polygon and without a neighbour within two body lengths yielded similar polygon reductions as observed in real fish groups of the surface molly (Table 4B, Fig. 3). In contrast, surface mollies did not change their behaviour from fission–fusion to schooling as indicated by our analysis of swimming polarisation. While surface mollies generally appeared to have a higher polarisation than cave mollies, surface mollies did not change their degree of polarisation when the density decreased (mean sum of unit vectors; surface mollies: G12a = 0.48, G6 = 0.48, G12b = 0.48; cave mollies: G12a = 0.37, G6 = 0.36, G12b= 0.33).

## Discussion

We investigated two populations of the Atlantic molly that colonised adjacent, but vastly different habitats. While surface-dwelling mollies can be found in sulfidic springs with high levels of bird predation, the cave environment inhabited by the cave molly can be assumed to be free of visually hunting predators. In combination with hampered visual sensing in the cave, we predicted that cave-dwelling mollies may not be as social as their surface-dwelling counterparts. Furthermore, cave mollies may not possess the presumably adaptive ability to keep their social dynamics constant across different densities. In congruence with our predictions, we found cave mollies to spent less time being social than surface mollies. This was a consequence of surface mollies being less likely to leave a current social partner and to stop being social than cave mollies. There was also a trend for less partner switching in surface mollies in groups of 12 individuals compared to cave mollies. Cave and surface mollies also differed in the probability to become social after a phase of being alone (*P*_a→s_). When fish densities were experimentally reduced, surface mollies unlike cave mollies were able to maintain their fission–fusion dynamics, e.g., kept similar probabilities of switching from social to asocial periods. Surface mollies achieved this by a reduction in the size of a convex polygon formed by the group as density decreased.

Given the unusually high bird predation outside the cave (Riesch et al., 2010), moving away from partners and, to a lesser extent switching partners is presumably risky and this selection pressure should promote the formation of stronger social bonds between individuals. This is an aspect of our research that deserves further attention and has also been highlighted when comparing the social structure of high- and low-predation populations of the guppy, *P. reticulata* (Kelley et al., 2011). The surface mollies’ high percentage of time spent being social (81–92%) is comparable to the most extreme high-predation populations of guppies in Trinidad (Magurran & Seghers, 1991) and likely a results of their low probability to stop being social and the high probability to re-join a conspecific once alone.

In contrast, the fission–fusion dynamics of the cave mollies largely followed a null-model (with no social interactions). Magurran et al. (1992) reported that in a high-predation guppy-population (*P. reticulata*) shoaling was significantly reduced (by 2–2.5 times in males and females, respectively) after the fish had been introduced to a low-predation area and left there for over 50 years to reproduce and adapt. By comparison, shoaling behaviour seems to have disappeared completely in our cave mollies (despite the very high densities of fish with more than 200 individuals per square metre in some chambers. see Jourdan et al., 2014), and it is estimated that ancestral surface mollies entered this cave approximately 100 ka ago (Pfenninger et al., 2014). It is known from lab-reared cave mollies that they show shoaling behaviour under daylight conditions and are only asocial under low-light conditions (in contrast to surface mollies which maintain some shoaling behaviour under low-light conditions too, Bierbach et al., 2018). Further research on other populations that inhabit environments both inside and outside caves will be necessary to complete the picture, for example using the Mexican cave tetra (*Astyanax mexicanus*) and its surface-dwelling relatives (Protas et al., 2007; Panaram & Borowsky, 2005). Here it would be interesting to know whether these cave populations show similar properties of a “natural null model” (see Kowalko et al., 2013), although it is known that cave tetras are highly aggressive among each other (Yoshizawa, 2015) and may have developed much better non-visual sensing as they completely lost their eyes during the adaptation to the cave habitats (Jeffery, Strickler & Yamamoto, 2003).

The surface mollies displayed an ability to maintain their fission–fusion dynamics despite density changes which was not observed in cave mollies. Relatively little work has been done on the response of social networks to experimental perturbations. Simulated random removal of random individuals in killer whales (*Orcinus orca*) showed no strong effect on the network but targeted removal of females predicted network fragmentation (Williams & Lusseau, 2006). A similar result was obtained in a study with captive pig-tailed macaques (*Macaca nemestrina*) where removal of high-ranking individuals resulted in network fragmentation (Flack et al., 2006, see also Manno, 2008 for similar results). In contrast,  Mourier, Brown & Planes (2017) carried out targeted removals of blacktip sharks (*Carcharhinus melanopterus*) which showed no effect on network structure. In the forked fungus beetle, (*Bolitotherus cornutus*) social networks were found to remain stable after disturbances but changed through increasing interaction levels when not disturbed (Formica et al., 2017). The consequences of removals or introductions of individuals for group or population networks should also be relevant for conservation, management (Snijders et al., 2017) and animals in captivity (such as zoos; Kleinhappel et al., 2016).

The fact that the surface mollies, just like guppies (Wilson et al., 2014), can compensate for density changes to maintain their fission–fusion dynamics suggests that this particular level of dynamics possesses an important adaptive value which is presumably linked to anti-predator benefits and information exchange. So, with half the number of fish in the arena, how can surface mollies still keep similar probabilities to switch from social to asocial and vice versa under varying densities while simultaneously visiting most of the available space in the arena? A reduction of the used area in the arena was detectable in the low density treatment (21% reduction), but a much greater reduction (about 60%) would have been necessary to explain the observed compensation in the fission–fusion dynamics by the area usage hypothesis. The reduction in the polygon size spanned by the group was much greater than one would expect by a reduction in density alone. This suggests a rather simple mechanism for maintaining fission–fusion dynamics under changing density contexts. Such a reduction in polygon size could be achieved by fish moving back into the group when they are at the vertex of a convex polygon spanned by their neighbours that are outside the social range (in our case outside an eight cm radius, see Fig. 3). The theory of marginal predation (Hamilton, 1971) suggests that if predators attack the closest prey, then those on the edge of groups should experience greater risk (Bumann, Krause & Rubenstein, 1997; Morrell & Romey, 2008; Ioannou et al., 2019). Thus, a strong selection pressure for individuals to move back into the group, e.g., swim within the vertex polygon, is likely to be common in group-living animals (see Morrell, Ruxton & James, 2011).

We did not find any indication that the surface mollies switched from fission–fusion behaviour to schooling behaviour in response to being at a lower density. Also, the polarisation of surface mollies was much lower than for typical schooling behaviour observed in other poeciliid studies (showing values of 0.75 and higher, see Herbert-Read et al., 2012). Therefore, we conclude that increased polarisation was not responsible for the observed reduction in the polygon size in surface mollies.

Our study design was focused on social networks in semi-natural environments, for which we did not remove our study animals from their respective habitats. While this allowed us to test our predictions without major short-comings of purely laboratory-based experiments, we are aware that our study has some other weaknesses. (1) Due to the lack of other available surface/cave population pairs in that region, we could make our comparison for only one population inside and one outside a cave. (2) Logistical constrains at the field sites allowed us to only replicate our networks twice for surface and three times for cave mollies. (3) We are aware that our density reduction treatment may be biased due to order effects (always 12-6-12 order). Nevertheless, the densities used in our current experiment are comparable to those typically found in both habitats investigated: Jourdan et al. (2014) lists an average of 21.0 ± 5.0 individuals/m^2^ for the El Azufre river and 37.4 ± 4.8 individuals/m^2^ for cave chamber X (but much higher densities in the chambers closer to the cave entrance). Our 12 and 6 individual treatments reflect densities of 33.3 and 16.6 individuals/m^2^, respectively. We are therefore confident that our treatments simulate natural density conditions for the fish. (4) We recorded only short snapshots of 3 min per treatment. While longer observation periods would be desirable, our fine-scaled tracking analysis along with the particular strength of the Markov modelling approach to depict different components of the social behaviour of our study populations may help to compensate for this issue. Our study thus serve as a valid seed point for future research on this system that should include more within-population replicates as well as longer observation periods.

## Conclusions

Our study shows that cave and surface mollies differ in their fission–fusion dynamics especially when they have to deal with density changes. While cave mollies largely followed a null-model (with no social interactions), i.e., they showed reduced social encounters in the low density treatment, surface mollies were able to maintain their interaction probabilities even at low density. We propose that surface mollies can achieve this through a simple movement rule: fish move back into the group when they are at the vertex of a convex polygon spanned by their neighbours that are outside the social range. We interpret the found patterns as an evolutionary consequence of the vastly different predator regimes these populations experienced. However, the consequences of the complete lack of shoaling behaviour for the foraging ecology and mating behaviour in the cave molly are largely unknown. Future research, especially in comparison to other cave-dwelling animals may help to gain insights into how evolutionary processes led to the observed loss of shoaling behaviour and its consequences for the natural history of cave-dwelling species.

##  Supplemental Information

10.7717/peerj.8974/supp-1 Supplemental Information 1 Supplementary Information

10.7717/peerj.8974/supp-2 Table S1 Tracking data along with fish IDs

10.7717/peerj.8974/supp-3 Video S1 The experimental net cage Jens Krause is inspecting the experimental net cage inside the cueva del azufre, Tabasco, Mexico.

## Additional Information and Declarations

### Field Study Permissions

The following information was supplied relating to field study approvals (i.e., approving body and any reference numbers): Experiments reported in this study were carried out in accordance with the recommendations of “Guidelines for the treatment of animals in behavioural research and teaching” (published in Animal Behaviour 1997) and under the authorization of the Mexican government (DGOPA.09004.041111.3088, PRMN/DGOPA-003/2014, PRMN/DGOPA-009/2015, and PRMN/DGOPA-012/2017, issued by SAGARPA-CONAPESCA-DGOPA).

### Data Availability

The following information was supplied regarding data availability: The tracking data along with fish IDs is available in the Supplemental Files.
