# Supplementary material for: An interaction mechanism for the maintenance of fission–fusion dynamics under different individual densities
Source: PeerJ. 2020 May 14;8:e8974. doi: 10.7717/peerj.8974 (PMC7231501; doi:10.7717/peerj.8974)
Supplement: Supplemental Information 1 [file peerj-08-8974-s001.docx]

**Supplementary material for**

**An interaction mechanism for the maintenance of fission-fusion dynamics under different individual densities**

D. Bierbach, S. Krause, P. Romanczuk, J. Lukas, L. Arias-Rodriguez, J. Krause

**SI Text 1: Details on the Markov chain model for analysing fission-fusion dynamics.**

The data points for the construction of the model were collected by observing ‘behavioural states’ of focal individuals every ***t*** seconds. At each time point the focal individual can either be alone (no conspecific within a radius of ***d*** cm), denoted by the state *a*, or can have another individual *g* as nearest neighbour, denoted by the state *s_g_*. A focal individual is regarded as being social, if it is in state *s_g_* for some neighbour *g*. ‘Being social’ is not an explicit state in the model, but is implicitly defined by the set *s* = {*s_1_*, ..., *s_k_*}, where *k* is the number of potential neighbours. Following Wilson, Krause et al. (2014) we used the three probabilities

*P_leave_nn_* = P(state_n+1_ ≠ *s_g_* | state_n_ = *s_g_*).

*P_s_*_→_*_a_* = P(state_n+1_ = *a* | state_n_ ∈ *s*), and

*P_a_*_→_*_s_* = P(state_n+1_ ∈ *s* | state_n_ = *a*)

to construct a model that describes the transitions between these states. Here, *P_leave_nn_* denotes the probability of leaving the current nearest neighbour, and *P_s_*_→_*_a_* (*P_a_*_→_*_s_*) the probability that the focal individual will be alone (social) in the next state when it is currently social (alone). Figure S1 shows a graphical representation of this model. The probability *P_leave_nn_* is not explicitly shown in the figure. It is equal to 1−*P_retain_nn_* (and to *P_s_*_→_*_a_* + *P_new_nn_* ). Another probability, which can be derived from the model, is the probability *P_switch_nn_* of switching social partners given the individual stays social. It can be computed in the following way

*P_switch_nn_* = *P_new_nn_* / *P_s_*_→_*_s_*.

For our study we chose ***d*** = 8 cm and ***t*** = 5 s. We confirmed that our results were robust regarding other choices for ***d*** and ***t*** (Figure S2), using 6 cm and 10 cm as values for ***d***, and 3 s and 10 s as values for ***t***.

**SI Text 2: Details on the random walk simulation**

We simulated random walks using the simulation model described by Wilson, Krause et al. (2015), which was developed to investigate the effects of density changes in groups of guppies. The model uses a discrete-time simulation where between two successive time points an individual can either be moving or resting. At each time point, an individual can retain its state (moving or resting) with fixed probabilities *P_moving_* and *P_resting_*, respectively, or change it. A moving individual moves by a fixed distance *l* in a direction given by the individual’s heading *h*. Additionally, at each time point an individual can decide to change its heading with probability *P_change_heading_*. A new heading is computed by adding to the current heading a value α, which is drawn from a von Mises distribution (a circular analogue of the normal distribution) on the range (-π, +π) with concentration 1. As simulation ‘world’ we used a square. We prevented individuals from ‘leaving’ the simulation world by letting them choose a new heading (using the von Mises distribution), if necessary, until a movement in this direction of length *l* was within the square. By changing the number of individuals while keeping constant all other parameters of the movement simulation we determined the relative magnitude of changes to the measures under investigation (fission-fusion dynamics, area use, size of group polygon) caused by changing density.

**SI Text 3: Brief description of data sources and analysis used**

**Data sources**

**Experiment cave and surface mollies (Obs data)**

- 180s with 5fps sampling rate per individual and treatment (G12a-G6-G12b)
- 3 groups cave mollies, 2 groups surface mollies, 12 fish per group

**Random walk simulation (Sim data)**

- Individual based movement model as in Wilson et al. 2015, see SI Text S2
- Sampled as experimental data (180s, 5fps per individual)
- 10^4^ repeats

**Analysis**

**Fission-fusion dynamics**

- Markov-Chain-Model (see SI Text S1)
- **Input:** Data from Obs and Sim, nearest neighbor (8 cm radius) every 5s sampled
- **Output:** probabilities to change social state between time steps and overall % time being social
- Comparison among treatments in Obs and Sim data. Significant differences assumed when 95% CIs do not overlap (only ½ of data used to calculate CIs to account for simultaneous observations within groups)

**Exploring possible mechanisms of active density compensation**

- **Swimming distances (speed) in cave and surface-dwelling mollies:**
  - Input: Obs data; 180 s with 5fps sampling rate data, only those 6 individuals present in all treatments
  - Output: Mean individual swimming distance per treatment
  - Comparison among treatments : difference in mean swimming distance in Obs data compared to 0.025 percentile when individuals were randomly assigned to treatments (randomization test, 10^5^ repeats)
- **Area usage in cave and surface-dwelling mollies**
  - Input: Obs and Sim data: 180 s with 5fps sampling rate data, all individuals
  - Output: % of visited 15 x 15 cm squares in arena
  - Comparison: % area reduction observed from 12 to 6 indindividuals in Obs data
  - AND comparison of fission-fusion probabilities in Obs data with Sim and Sim (reduced area) data
- **Size of the polygons formed by the groups of cave and surface-dwelling mollies**
  - Input: Obs and Sim data: 180 s with 5fps sampling rate data, all individuals
  - Output: mean perimeter and mean area of the convex polygons
  - Comparison: % reduction from 12 to 6 individuals in Obs and Sim data.
  - **Movement Rule**
    - as in ‘Size of the polygons’ but comparison to Sim data with new movement rule implemented
- **Polarization**
  - Input: Obs data; every 3s sampled, only 6 individuals present in all treatments
  - Output: sum of the unit velocity vectors
  - Comparison among treatments: change observed from 12 to 6 individuals in Obs data

**Figure S1: Markov chain model of general fission-fusion behaviour.** A focal individual can either be social, i.e. with some neighbour *g*, denoted by *s_g_* or alone denoted by *a*.

**Figure S2**. Estimated model probabilities plus 95 % confidence intervals for cave (blue) and surface mollies (red) for different values of the model parameters *d* and *t*. With increasing values of *t* the confidence intervals become larger, because the number of data points decreases. The absolute values of the probabilities depend on the choice of *d* and *t*, but the relative differences among cave and surface molly values are highly consistent across the range of investigated *d* and *t*.

a)

d = 8 cm, t = 5 s


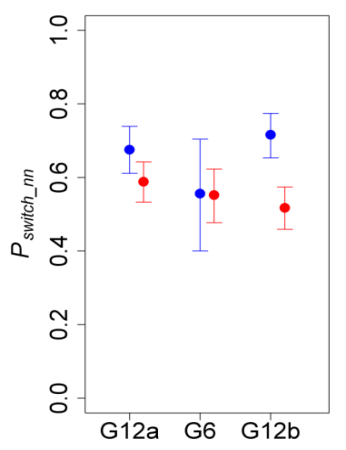

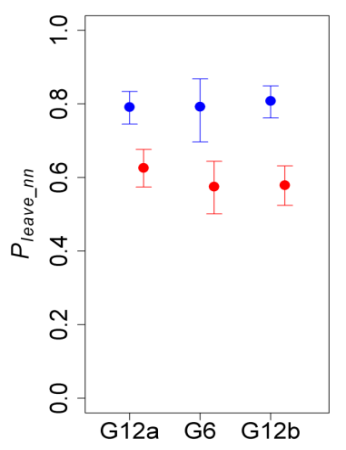

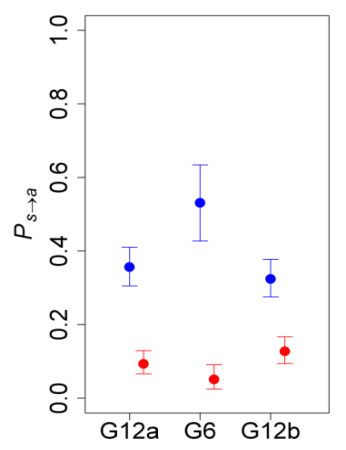

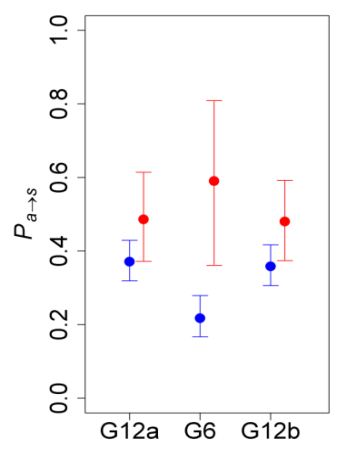


d = 6 cm, t = 3 s

b)


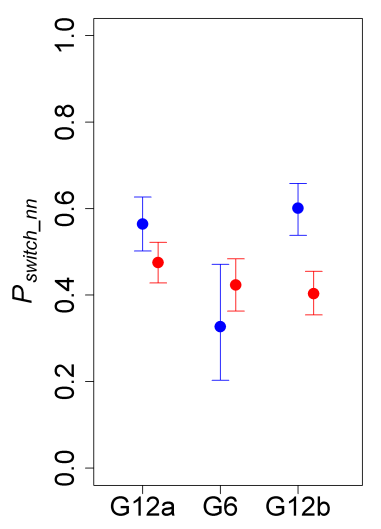

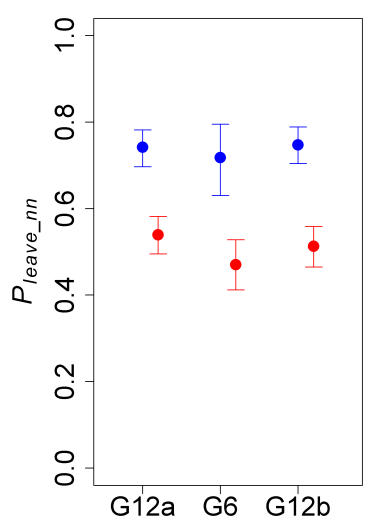

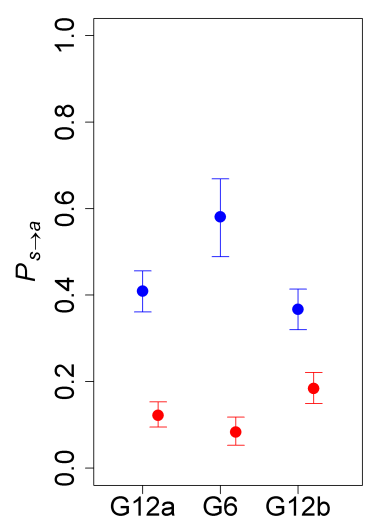

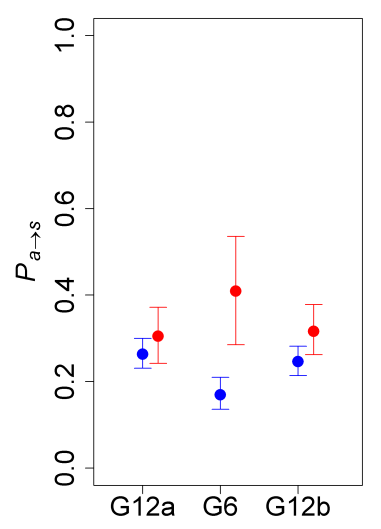


c)

d = 10 cm, t = 10 s


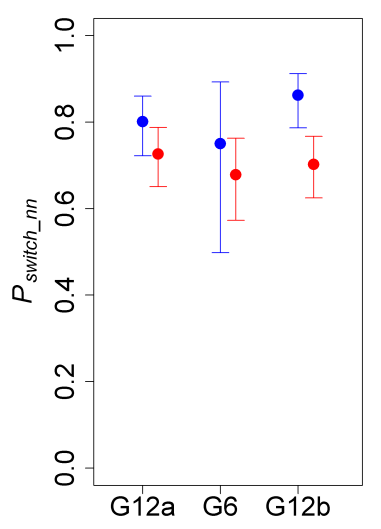

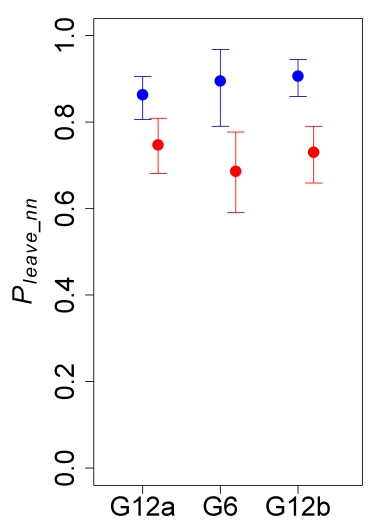

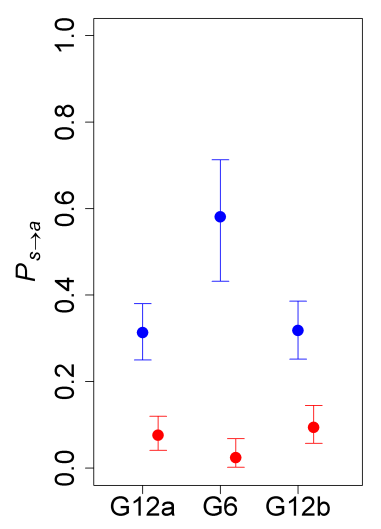

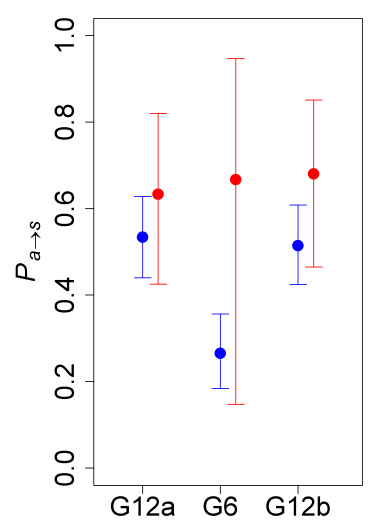


**SI Table 1: Tracking data along with fish IDs. The first 2 sheets list fish IDs, the following sheets XY- positions.**
